# The role of ventral and preventral organs as attachment sites for segmental limb muscles in Onychophora

**DOI:** 10.1186/1742-9994-10-73

**Published:** 2013-12-05

**Authors:** Ivo de Sena Oliveira, Noel N Tait, Ira Strübing, Georg Mayer

**Affiliations:** 1 Animal Evolution and Development, Institute of Biology, University of Leipzig, Talstraße 33, D-04103, Leipzig, Germany; 2 Department of Biological Sciences, Macquarie University, Sydney, New South Wales, 2109, Australia

**Keywords:** Apodeme, *Delta*, Limb musculature, *Notch*, Peripatidae, Peripatopsidae, Velvet worm

## Abstract

**Background:**

The so-called ventral organs are amongst the most enigmatic structures in Onychophora (velvet worms). They were described as segmental, ectodermal thickenings in the onychophoran embryo, but the same term has also been applied to mid-ventral, cuticular structures in adults, although the relationship between the embryonic and adult ventral organs is controversial. In the embryo, these structures have been regarded as anlagen of segmental ganglia, but recent studies suggest that they are not associated with neural development. Hence, their function remains obscure. Moreover, their relationship to the anteriorly located preventral organs, described from several onychophoran species, is also unclear. To clarify these issues, we studied the anatomy and development of the ventral and preventral organs in several species of Onychophora.

**Results:**

Our anatomical data, based on histology, and light, confocal and scanning electron microscopy in five species of Peripatidae and three species of Peripatopsidae, revealed that the ventral and preventral organs are present in all species studied. These structures are covered externally with cuticle that forms an internal, longitudinal, apodeme-like ridge. Moreover, phalloidin-rhodamine labelling for f-actin revealed that the anterior and posterior limb depressor muscles in each trunk and the slime papilla segment attach to the preventral and ventral organs, respectively. During embryonic development, the ventral and preventral organs arise as large segmental, paired ectodermal thickenings that decrease in size and are subdivided into the smaller, anterior anlagen of the preventral organs and the larger, posterior anlagen of the ventral organs, both of which persist as paired, medially-fused structures in adults. Our expression data of the genes *Delta* and *Notch* from embryos of *Euperipatoides rowelli* revealed that these genes are expressed in two, paired domains in each body segment, corresponding in number, position and size with the anlagen of the ventral and preventral organs.

**Conclusions:**

Our findings suggest that the ventral and preventral organs are a common feature of onychophorans that serve as attachment sites for segmental limb depressor muscles. The origin of these structures can be traced back in the embryo as latero-ventral segmental, ectodermal thickenings, previously suggested to be associated with the development of the nervous system.

## Background

The body plan of the Onychophora, the putative sister group of the arthropods, displays a combination of segmental and non-segmental features [1,2]. While parts of the integument, muscular and nervous systems do not show any segmentation, segmental organisation is evident, such as in the arrangement of nephridia [3,4], ostia of the heart [5,6], crural glands [7,8], limbs with their muscles, apodemes and nerves [1,9-12], and the so-called ventral organs [13-15]. The ventral organs are arguably one of the most controversial structures in Onychophora, as their development, anatomy, relationship to other structures and consequently their function remain unclear [1,16-22]. Initially, the ventral organs were described as embryonic segmental, paired thickenings of the ventro-lateral ectoderm, which arise late in onychophoran development [23-25], but the term ventral organ has also been applied to segmental, cuticular structures that occur mid-ventrally between each leg pair in adult onychophorans [7,8,13-15,26-28]. Whether the adult ventral organs are remnants of the embryonic thickenings [1,8,17,23,24] or whether these thickenings are transitory structures that disappear completely during development [19-21] is controversial.

Moreover, the role of the embryonic ventral organs is ambiguous [29], as they have been regarded either as rudiments of an ancient locomotory system comparable to the ventral ciliated areas of annelids [16], or as segmental anlagen of the nervous system or as rudimentary ganglia [19-22,30]. Based on the last assumption, the embryonic ventral organs of onychophorans have been homologised with homonymous structures found in embryos of chelicerates and myriapods, in which they appear as paired, segmental epithelial vesicles that are involved in neurogenesis and incorporated into the segmental ganglia during development [2,19,20,31,32]. However, subsequent studies have demonstrated that the ventral organs are not associated with the nervous system in the onychophoran embryo as they arise after the presumptive nerve cords have formed [17,29]. Thus, the homology of the onychophoran ventral organs to the homonymous structures in chelicerates and myriapods is unlikely [1,17].

To further complicate matters, additional structures called “preventral organs” have been described from adults of a number of species of onychophorans [7,13-15]. These structures are morphologically similar to the ventral organs, but they are smaller and located anterior to each ventral organ [14,15]. Currently, neither the embryonic fate nor the relationship of the preventral organs to the embryonic and adult ventral organs of onychophorans is known. To clarify whether the ventral and preventral organs are a common feature of Onychophora, we analysed the anatomy of eight species of velvet worms, including representatives of the two major onychophoran subgroups, Peripatidae and Peripatopsidae. In addition, we have documented the embryonic fate of the ventral and preventral organs in embryos of *Euperipatoides rowelli* using *in situ* hybridization, histochemistry and immunocytochemical methods, in conjunction with confocal microscopy, to gain insights into their function.

## Results

### Position and structure of the ventral and preventral organs

**Figure 1:**
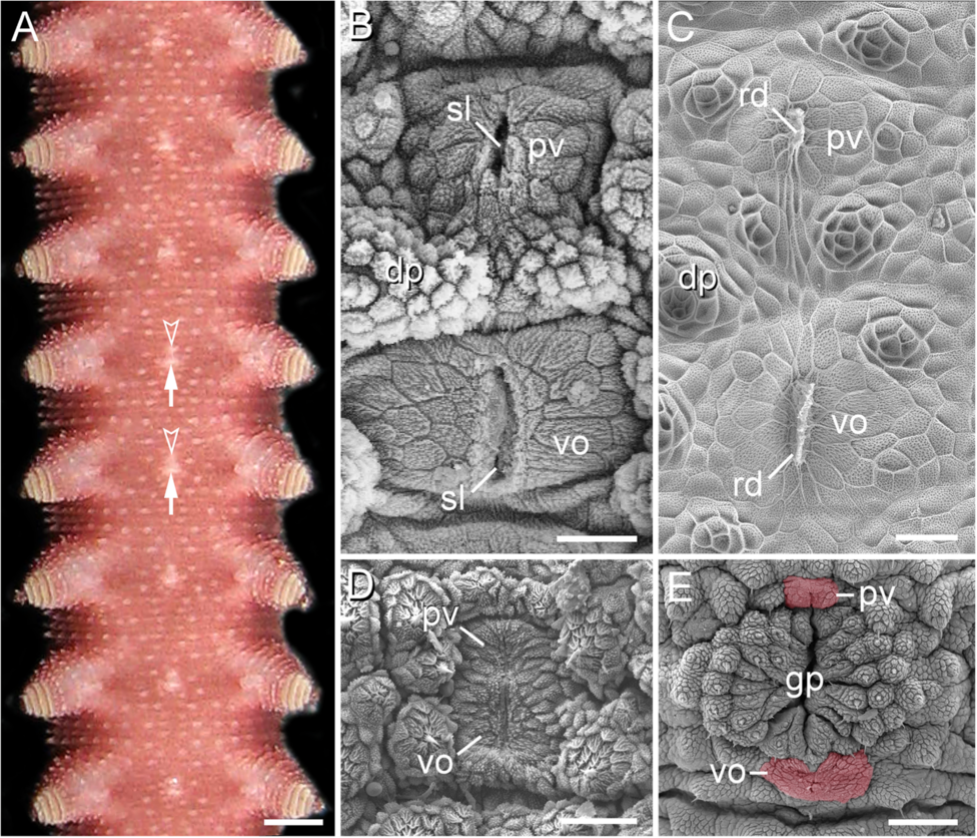
**Position and structure of the ventral and preventral organs in adult onychophorans.** Light micrograph **(A)** and scanning electron micrographs **(B–E)**. Anterior is up in all images. **(A)** Overview of the ventral body surface in an anesthetised specimen of *Epiperipatus* sp. 1 (Peripatidae)*.* Note the ventral (arrows) and preventral (arrowheads) organs, which appear as bright segmental spots along the ventral midline. **(B)** Ventral and preventral organs in *Epiperipatus biolleyi* (Peripatidae), which are widely separated, as in all other peripatids. **(C)** Inner surface of a moulted cuticle of *Principapillatus hitoyensis* (Peripatidae) showing the median sclerotized ridges of the ventral and preventral organs. **(D)** Ventral and preventral organs in the peripatopsid *Metaperipatus inae* that, in contrast to the peripatids, appear as a unitary structure. **(E)** Genital region in *E. biolleyi.* Note that the genital opening/pad lies between the ventral and preventral organs (labelled by an artificial colour), as in other species of onychophorans studied (cf. Additional file 1). Abbreviations: dp, dermal papilla; gp, genital opening/pad; pv, preventral organ; rd, median ridge; sl, median slit; vo, ventral organ. Scale bars: 1 mm **(A)**, 25 μm **(B)**, 30 μm **(C)**, 50 μm **(D)**, and 100 μm **(E)**.

**Figure 2:**
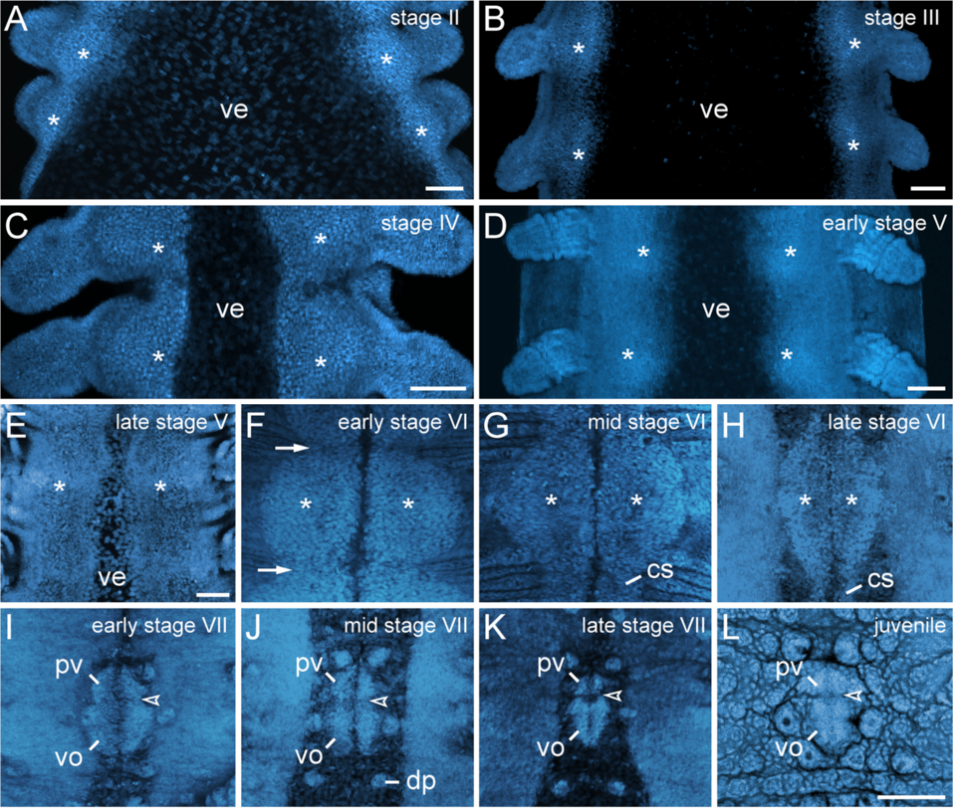
**Morphogenesis of the ventral and preventral organs.** Embryos of *Euperipatoides rowelli* (Peripatopsidae) at successive developmental stages labelled with the DNA marker Bisbenzimide **(A–K)** and RedDot^TM^2 **(L)**. Confocal laser-scanning micrographs. Anterior is up in all images. The asterisks in **A–H** indicate the paired ectodermal thickenings that become the anlagen of the ventral and preventral organs. **(A)** Stage II embryo. **(B)** Stage III embryo. **(C)** Stage IV embryo. **(D)** Early stage V embryo. **(E)** Late stage V embryo. **(F)** Early stage VI embryo. At this stage, the ectodermal thickenings achieve their largest size. They become distinct from the remaining ectoderm and are delineated from each other by transverse furrows (arrows) along the main body axis. **(G)** Mid stage VI embryo. **(H)** Late stage VI embryo. Note the persisting cell strands that connect the thickenings along the body. **(I)** Early stage VII embryo. At this stage, the cell strands have degenerated and a border between the anlagen of the ventral and preventral organs (arrowheads in **I**–**L**) starts forming. **(J)** Mid stage VII embryo. **(K)** Late stage VII embryo. **(L)** Newborn juvenile specimen. Note that the paired anlagen of the ventral and preventral organs have fused medially, but their paired nature is still recognisable. Abbreviations: cs, cell strand; dp, dermal papilla; pv, preventral organ anlage; ve, ventral extraembryonic tissue; vo, ventral organ anlage. Scale bars: 100 μm **(A–E, L)**. The images **F**–**L** are to scale (scale bar provided in **L**).

The ventral body surface of onychophorans typically shows mid-ventral, segmentally repeated bright spots between each leg pair (Figure 1A). Scanning electron microscopy in representatives of Peripatidae reveals that each spot consists of a large, paired, roundish posterior structure (=ventral organ) and a similar, albeit smaller, paired anterior structure (=preventral organ) (Figure 1B). Externally, the paired nature of each ventral and preventral organ is evident by a median, longitudinal slit (Figure 1B). Examination of the internal structure of moulted skins reveals that each slit is formed by an invaginated cuticle, which gives rise to an apodeme-like, longitudinal ridge (Figure 1C). While the ventral and preventral organs are widely separated from each other in representatives of Peripatidae (Figure 1B), their cuticle forms a unitary structure in species of Peripatopsidae (Figure 1D, E). However, even in the peripatopsids, the ventral and preventral organs are recognisable as separate structures at the cellular level as well as during embryogenesis (Figure 2A–L). In both onychophoran subgroups, the separation of these two structures is most obvious in the genital segment, in which the genital pad is located between the preventral and ventral organs (Figure 1E; Additional file 1).

### Morphogenesis of the ventral and preventral organs

During embryogenesis of the peripatopsid *Euperipatoides rowelli*, the ventral and preventral organs arise from single paired, segmental, ectodermal thickenings (Figure 2A–L). DNA labelling of embryos at subsequent developmental stages revealed that the thickenings appear early in development as segmentally repeated undulations of the ventrolateral ectoderm (Figure 2A–E). Initially, the thickenings of each body side are widely separated from each other by the ventral extraembryonic tissue, which is reduced during development, while the ventrolateral ectoderm of both sides fuses along the ventral midline (Figure 2A–F). After this fusion, the unitary anlagen of the ventral and preventral organs are recognisable as paired segmental structures that are separated by repeated transverse furrows along the body (arrows in Figure 2F). At this developmental stage, the paired thickenings, i.e., the anlagen of the ventral and preventral organs, occupy nearly the entire ventral body surface (Figure 2F, G).

**Figure 3:**
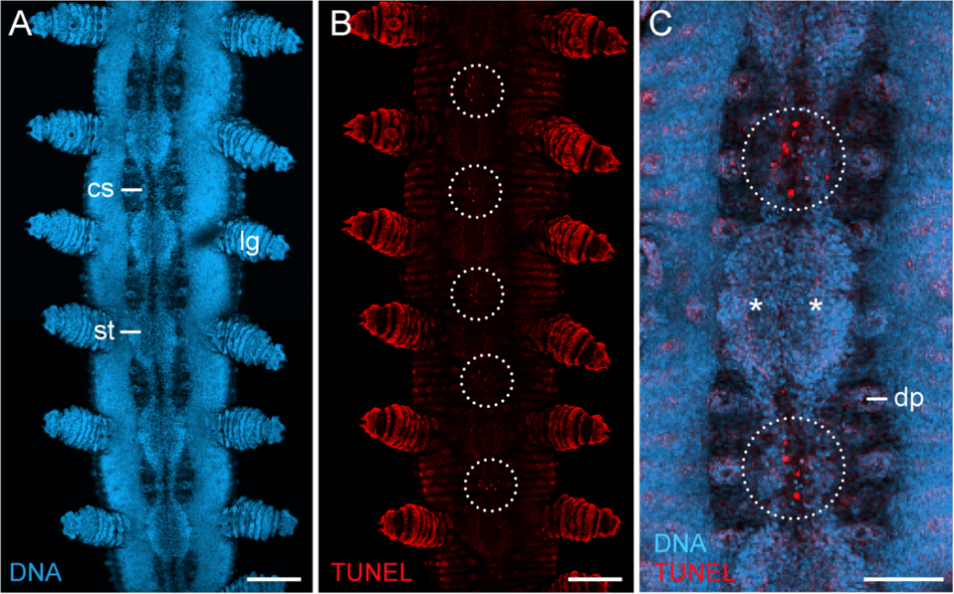
**Apoptotic cells during degeneration of segmental thickenings.** Confocal laser-scanning micrographs from a late stage VI embryo of *Euperipatoides rowelli*  (Peripatopsidae). Same embryo as in Figure 2H. Anterior is up in all images. **(A)** Overview of the ventral body surface labelled with the DNA marker Bisbenzimide. Note the persisting, paired cell strands that connect the segmental thickenings along the body. **(B)** Same embryo labelled with a cell death detection kit (Terminal deoxynucleotidyl transferase dUTP nick end labelling – TUNEL). Doted circles indicate areas in the interpedal regions, where numerous apoptoses are seen. **(C)** Detail of the same embryo showing a merged image of DNA and TUNEL labelling. Dotted circles indicate areas with numerous apoptotic cells. Asterisks indicate the paired segmental thickenings (=anlagen of the ventral and preventral organs). Abbreviations: cs, cell strand; dp; developing dermal papilla; lg, presumptive leg; st, segmental thickening. Scale bars: 200 μm **(A, B)**, and 100 μm **(C)**.

As development proceeds, the anlagen of the ventral and preventral organs decrease in size and numerous apoptoses occur in the interpedal regions, i.e., between subsequent leg pairs (Figures 2G–L and 3A–C). In the initial phases of this degeneration process, the anlagen of the ventral and preventral organs of adjacent segments remain connected along the body by thin, paired cellular strands (Figures 2G, H and 3A, C), but these strands disappear later in development (Figure 2I–L). After their disappearance, each remaining unitary anlage splits into a smaller anterior (=anlage of the preventral organ) and a larger posterior accumulation of cells (=anlage of the ventral organ), thus resulting in a double-paired ectodermal structure situated between each leg pair (Figure 2I–K). The anterior paired structure persists as the preventral organ, whereas the posterior paired structure is retained as the ventral organ in post-embryonic stages (Figure 2L).

### Expression of *Delta* and *Notch* in the anlagen of the ventral and preventral organs

**Figure 4:**
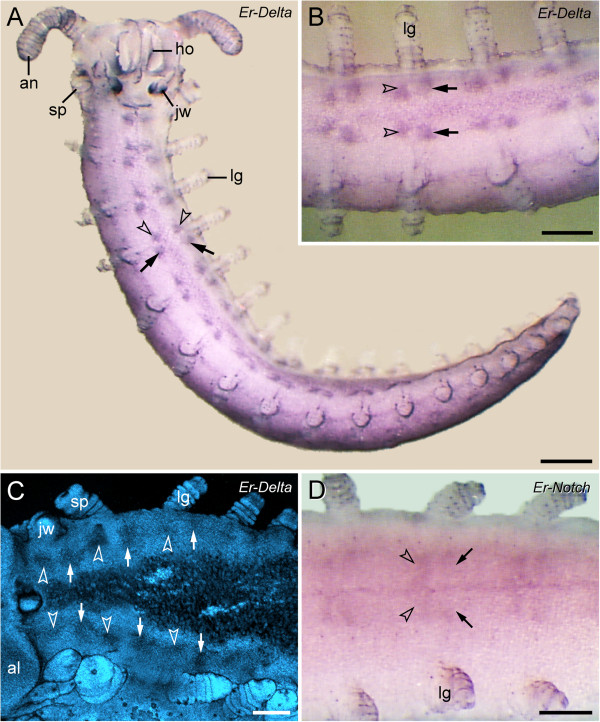
**Expression pattern of *Delta *and *Notch *homologs in embryos of the peripatopsid *Euperipatoides rowelli.*** Light micrographs **(A, B, D)** and confocal laser micrograph **(C)**. Anterior is left in **B**–**D**. Note the double-paired expression pattern of both genes along the ventral body surface, with a larger pair of posterior domains (arrows) and a smaller pair of anterior domains (arrowheads) in each body segment. **(A)** Overview of a late stage V embryo showing the expression pattern of *Er-Delta*. **(B)** Detail of the expression pattern of *Er-Delta* in the same embryo. **(C)** Expression of *Er-Delta* in a stage IV embryo, counterstained with the DNA marker Bisbenzimide. Note that the double-paired expression pattern of *Er-Delta* is already evident at this developmental stage. Also note that *Er-Delta* is expressed in a similar pattern in the jaw segment, whereas the fate of the ventral and preventral organs in this segment remains unclear. **(D)** Expression of *Er-Notch* in an early stage VI embryo. A weak expression is seen in a double-paired pattern, which is similar to the expression pattern of *Er-Delta.* Abbreviations: al, antennal lobe; an, presumptive antenna; ho, anlage of the hypocerebral organ; jw, presumptive jaw; lg, presumptive leg; sp, presumptive slime papilla. Scale bars: 400 μm **(A)**, and 200 μm **(B–D)**.

Our expression data of the *Delta* and *Notch* homologs in embryos of *Euperipatoides rowelli* revealed that the genetic differentiation of each unitary anlage into two separate structures, i.e., the presumptive ventral and preventral organs, precedes their morphological subdivision (cf. Figures 2A–L and 4A–D). According to our data, *Er-Delta* and *Er-Notch* are expressed in two paired domains between each pair of presumptive limbs in embryos of developmental stages, at which the anlagen of the ventral and preventral organs are not yet distinguishable as segmental structures (Figure 4A–D; cf. Figure 2D, E). In each segment, the anterior paired domain is clearly smaller than the posterior paired domain, thus corresponding in position, size and shape with the double-paired anlagen of the ventral and preventral organs (Figures 2I–L and 4B, D).

### No evidence for a relationship of the ventral and preventral organs to the nervous system

**Figure 5:**
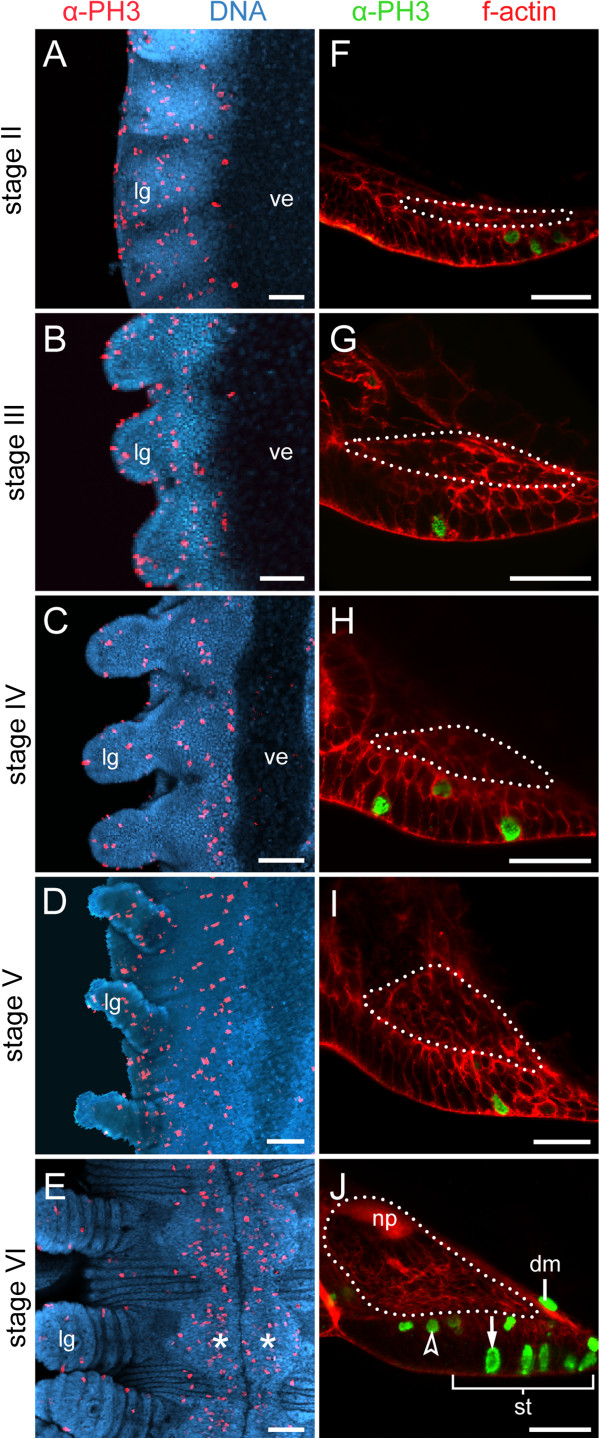
**Cell division pattern during the development of ectodermal thickenings.** Confocal laser-scanning micrographs. Embryos of *Euperipatoides rowelli* (Peripatopsidae) at successive developmental stages. **(A–E)** Cell divisions along the ventral body surface in embryos double-labelled with a DNA marker (Bisbenzimide; blue) and an anti-phospho-histone H3 antibody (red). Anterior is up. Note that cell divisions mainly occur within the segmental thickenings (=anlagen of the ventral and preventral organs) at stage VI (in **E**), whereas they occur in a random fashion along the body before this stage **(A–D)**. Note also that there are only a few dividing cells in the extraembryonic tissue. Asterisks (in **E**) indicate paired segmental thickenings. **(F–J)** Cross sections of embryos at corresponding stages double-labelled with phalloidin-rhodamine (f-actin; red) and an anti-phospho-histone H3 antibody (green). Note that the establishment of the nerve cord (dotted line) precedes the formation of segmental thickenings. Note also that the immigrated dividing neural progenitor cells (arrowhead in **J**) differ in shape and size from superficial dividing cells in the segmental thickening (arrow in **J**). Abbreviations: dm, dividing mesodermal cell; lg, leg anlage; np, nerve cord neuropil; st, segmental thickening (=anlage or the ventral and preventral organs); ve, ventral extraembryonic tissue. Scale bars: 100 μm **(A–E)**, and 50 μm **(F–J)**.

Our histochemical and immunocytochemical data provide no evidence for a developmental relationship of the ventral and preventral organs to the nervous system in embryos of *Euperipatoides rowelli*. A differentiation of the ectoderm into an internal layer of cells (neuronal precursors) and a superficial layer of remaining cells (epidermis, including the future ventral and preventral organs) occurs early in development, when no delimited anlagen of the ventral and preventral organs are as yet distinguishable (Figure 5A, F). At this developmental stage, the cells divide in a random fashion in the internal and superficial ectodermal cell layers (Figure 5A, F). This pattern persists until late in development (Figure 5B–D, G–I) but changes when the nerve cord neuropils have formed (Figure 5E, J). After neuropil formation, the unitary anlagen of the ventral and preventral organs have become delineated along the body and show a higher number of cell divisions than anywhere else in the ventral ectoderm (Figure 5E, J). Notably, the dividing cells in each anlage of the ventral and preventral organs have an elongated shape and are larger than the dividing neuronal precursors in the internal ectodermal layer (Figure 5J).

**Figure 6:**
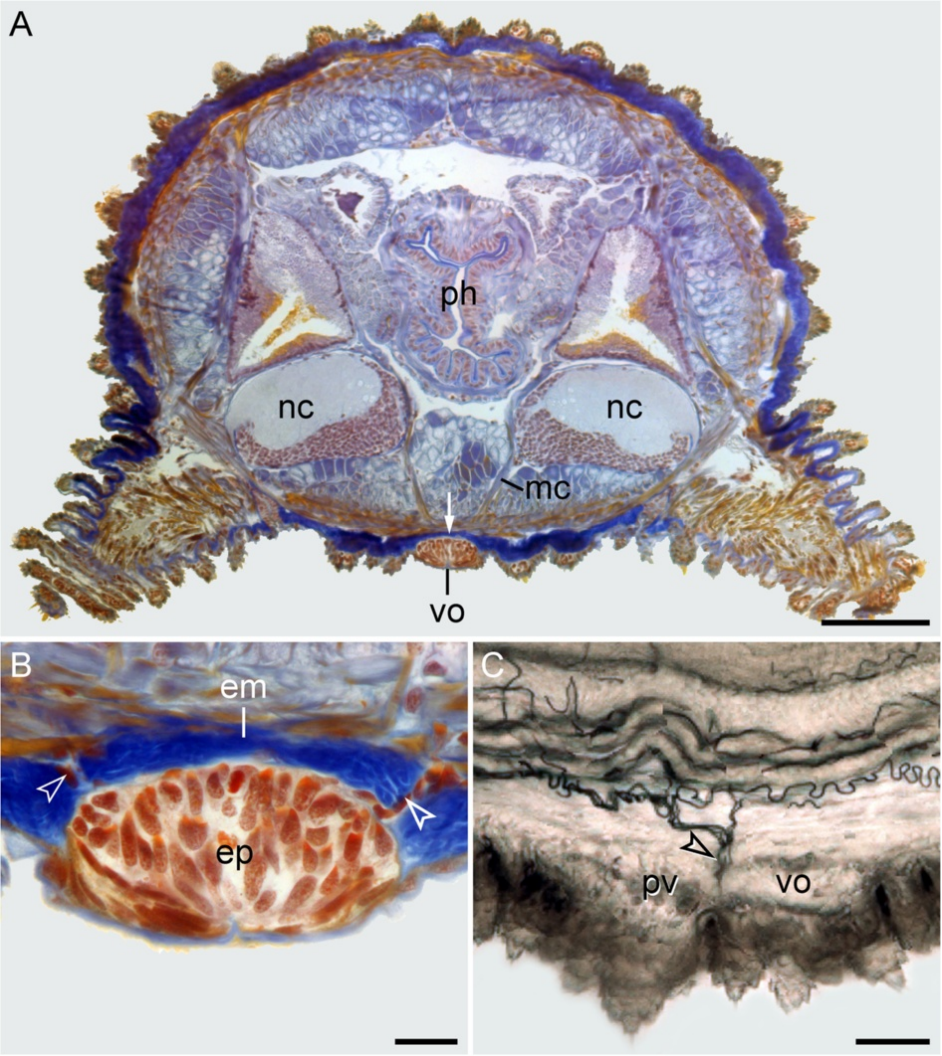
**Position and histology of the ventral and preventral organs in adult onychophorans.** Light micrographs. Dorsal is up in all images. **(A)** Histological cross-section (Azan staining) at the level of a ventral organ in *Metaperipatus blainvillei* (Peripatopsidae). Note the thick layer of extracellular matrix (arrow) separating the ventral organ from the remaining tissues. **(B)** Detailed view of a ventral organ in *Metaperipatus blainvillei*. Arrowheads point to bundles of tracheal tubes that cross the extracellular matrix in the vicinity of the ventral organ. **(C)** Sagittal Vibratome section at the level of the ventral and preventral organs in *Epiperipatus* sp. 2 (Peripatidae). Anterior is left. Note the bundles of tracheal tubes (arrowhead) that open to the exterior next to the ventral and preventral organs. Abbreviations: em, extracellular matrix; ep, epidermis of the ventral organ; mc, median commissure; nc, nerve cords; ph, pharynx; pv, preventral organ; vo, ventral organ. Scale bars: 200 μm **(A)**, 25 μm **(B)**, and 50 μm **(C)**.

Corresponding to these developmental data, no anatomical relationship of the ventral and preventral organs to the nervous system is evident in adult onychophorans. In post-embryonic stages and adults of the eight species studied, the ventral and preventral organs persist as epidermal structures, which are covered by a thick cuticle (Figures 1A–E, 2L and 6A, B). Notably, the ventral and preventral organs are clearly separated from the nervous system, musculature and other internal organs by a thick layer of extracellular matrix (Figure 6A, B). Although a few histological sections of adult specimens of *Metaperipatus blainvillei* display cellular strands that cross the extracellular matrix in the vicinity of the ventral/preventral organs (arrowheads in Figure 6B), our through-light micrographs of Vibratome sections from *Metaperipatus blainvillei*, *Principapillatus hitoyensis* and *Epiperipatus* sp. 2 show that these strands are tracheal tubes (Figure 6C; Additional files 2 and 3). The tracheal tubes that supply nerve cords and other internal structures usually take a course towards each nerve cord by following the median and ring commissures (Additional files 2 and 3). Some tracheal tubes commonly open to the exterior next to or between the ventral and preventral organs and these tracheal tubes are the only structures accompanied by nuclei that cross the extracellular matrix layer (Figure 6B, C; Additional files 2 and 3).

**Figure 7:**
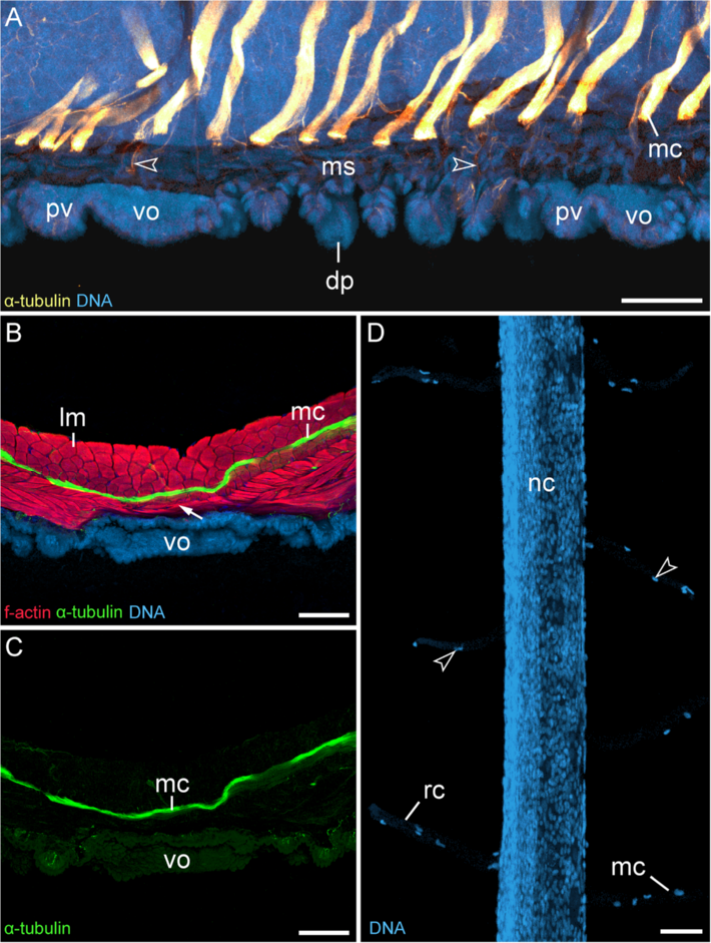
**Median commissures and their spatial relationship to the ventral and preventral organs.** Confocal laser-scanning micrographs of *Euperipatoides rowelli* (Peripatopsidae). **(A)** Sagittal section of a newborn juvenile, double-labelled with an anti-acetylated α-tubulin antibody (glow-mode) and a DNA marker (RedDot^TM^2; light-blue). Anterior is left and dorsal is up. Note that the serially repeated median commissures are not connected to the ventral and preventral organs but are spatially separated from them. Arrowheads point to single nerve fibres that innervate the dermal papillae. **(B)** Vibratome cross section of ventral body wall from an adult triple-labelled with an anti-acetylated α-tubulin antibody (green), a DNA marker (RedDot^TM^2; blue) and an f-actin marker (phalloidin-rhodamine; red). Dorsal is up. Note the layers of diagonal and ring musculature between the ventral organ and the median commissure (arrow). **(C)** Same section as in B showing only the anti-acetylated α-tubulin immunolabelling. Note the lack of nerve fibres or any connections between the ventral organ and the median commissure. **(D)** Detail of a dissected nerve cord from an adult specimen labelled with the DNA marker SYBR^®^ Green showing “glial” cells that accompany the ring commissures and the median commissures (arrowheads). Lateral is left. Abbreviations: dp, dermal papilla; lm, longitudinal musculature; mc, median commissure; ms, musculature; nc, nerve cord; pv, preventral organ; rc, ring commissure; vo, ventral organ. Scale bars: 50 μm **(A)**, 100 μm **(B, C)**, and 50 μm **(D)**.

In contrast to the tracheal tubes, median commissures, ring commissures and the leg nerves, which are all accompanied by “glial” cells (Figure 7D; Additional file 4), do not cross the extracellular matrix layer (Figure 7A–C). Single nerve fibres extend from each median commissure to supply the musculature and the dermal papillae (arrowheads in Figure 7A; Additional file 5), but these fibres are not accompanied by any nuclei or cell bodies. Hence, they do not correspond to the cell strands, which are seen occasionally next to the ventral organs in histological sections (cf. Figure 6B). Despite the impression in low magnification micrographs that the nerve cords connect to the ventral and preventral organs via the median commissures (Figure 6A), our immunocytochemical data show that each commissure passes above the circular and diagonal muscle layers to the contralateral body side (Figure 7A–C). Moreover, we find no spatial relationship in the arrangement of the median commissures and the ventral/preventral organs, as the median commissures are repeated along the entire body, including the interpedal regions, whereas the ventral and preventral organs are lacking in the interpedal regions (Figure 7A; Additional file 5).

### Musculature associated with the ventral and preventral organs

**Figure 8:**
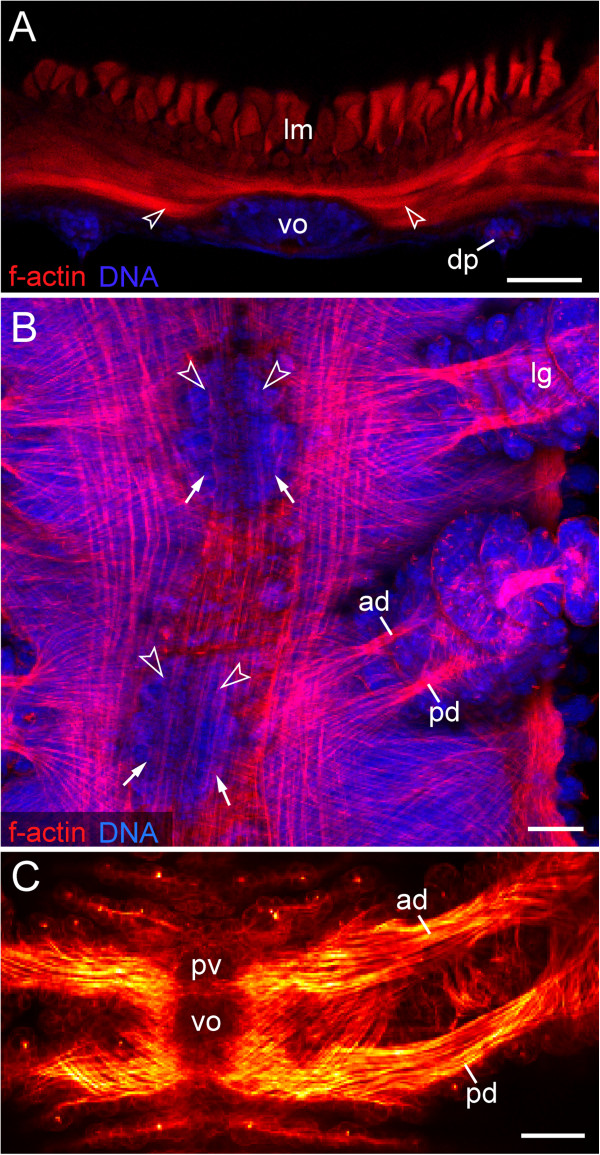
**Leg musculature associated with the ventral and preventral organs.** Confocal laser-scanning micrographs of *Euperipatoides rowelli* (Peripatopsidae). **(A)**  Vibratome cross-section of an adult specimen, double-labelled with phalloidin-rhodamine (f-actin; red) and a DNA marker (Bisbenzimide; blue) showing the prominent musculature associated with the ventral organ. Dorsal is up. Arrowheads point to ventral leg depressor muscles that attach to the ventral organ. **(B)** Musculature in an early stage VII embryo in ventral view (same embryo as in Figure 2I), double-labelled with phalloidin-rhodamine (f-actin; red) and a DNA marker (Bisbenzimide; blue). Anterior is up. Note that the presumptive anterior and posterior limb depressor muscles extend to the anlagen of the ventral (arrows) and preventral organs (arrowheads). **(C)** Limb musculature in a newborn juvenile in ventral view, labelled with phalloidin-rhodamine (f-actin; glow scale). Anterior is up. Note the anterior leg depressor, which connects to the preventral organ, whereas the posterior leg depressor attaches to the ventral organ. Abbreviations: ad, anterior leg depressor muscle; dp, dermal papilla; lg, presumptive leg; lm, longitudinal musculature; pd, posterior leg depressor muscle; pv, preventral organ; vo, ventral organ. Scale bars: 50 μm.

Vibratome sections of adult specimens of *Euperipatoides rowelli* labelled with an f-actin and a DNA marker revealed paired ventral muscles attached to each ventral organ (Figure 8A). The same labelling applied to embryos and post-embryonic stages shows that these are paired extrinsic leg muscles, i.e., the anterior and posterior leg depressors that attach medially to each preventral and ventral organ (Figure 8B, C). Each pair of the anterior depressors is associated with the preventral organ, whereas each pair of the posterior depressors is attached to the ventral organ (Figure 8C). This is also seen in confocal z-series, which revealed four attachment sites of leg depressor muscles in each leg-bearing segment (Additional file 6). Notably, a similar situation is found in the segment of the slime papillae that are modified limbs of the third body segment.

**Figure 9:**
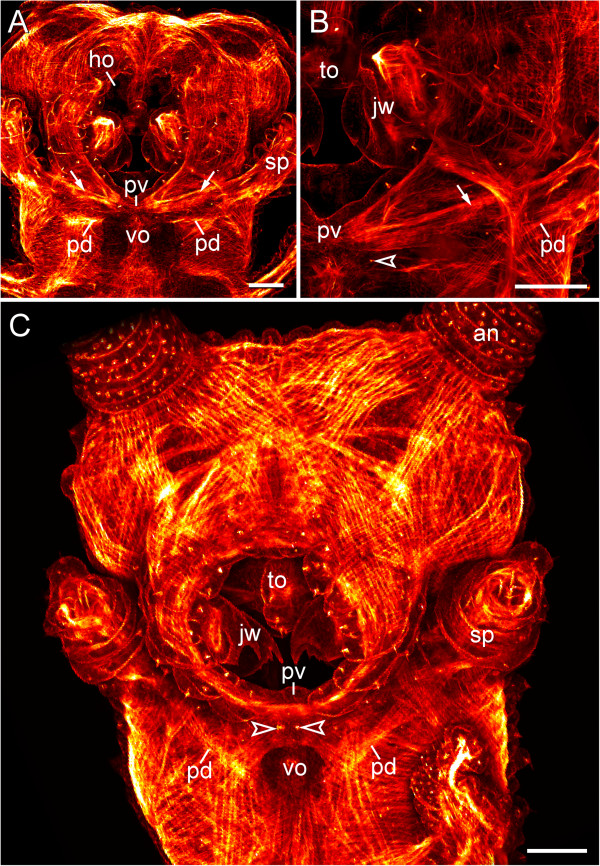
**Musculature of the slime papillae associated with the ventral and preventral organs. ** Confocal laser-scanning micrographs of embryos of *Euperipatoides rowelli* (Peripatopsidae) labelled with phalloidin-rhodamine (f-actin; glow scale). Anterior is up in all the images. **(A)** Overview of the musculature of the head in a stage VI embryo in ventral view. Arrows indicate the anterior depressor muscles of the slime papillae. **(B)** Detail from the same embryo as in **A** showing the attachment of the anterior depressor muscle (arrow) to the anlage of the preventral organ. Note the sensilla of the developing last pair of lips (arrowhead) situated posteriorly to the preventral organ. **(C)** Overview of the musculature of the head in a late stage VII embryo in ventral view. At this stage, the ventral and preventral organs of the slime papilla segment have been separated by the posterior-most pair of lip papillae (arrowheads). Note the posterior pair of depressor muscles of the slime papillae that are attached to the corresponding ventral organ situated posterior to the mouth. Abbreviations: an, presumptive antenna; ho, anlage of the hypocerebral organs; jw, presumptive jaws; pd, posterior depressor muscle of the slime papilla; pv, anlage of the preventral organ; sp, presumptive slime papilla; to, presumptive tongue; vo, anlage of the ventral organ. Scale bars: 100 μm.

**Figure 10:**
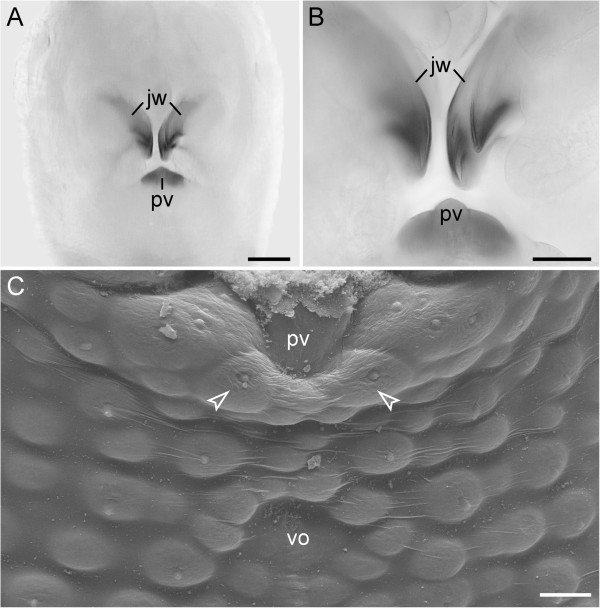
**Sclerotized mouth structures in embryos of the neotropical Peripatidae.** Light and scanning electron micrographs of flexed stage embryos of representatives of Peripatidae (posture stage according to Walker and Campiglia [33]). Anterior is up. **(A)** Head of an embryo of *Principapillatus hitoyensis* in ventral view. Note the scletotized plate posterior to the jaws, which corresponds in position with the preventral organ of the slime papilla segment that has migrated into the definitive mouth cavity. **(B)** Detailed view of the jaws and the sclerotized plate from the same embryo as in **A**. **(C)** Head of an embryo of *Plicatoperipatus jamaicensis* in ventral view. Arrowheads point to the posterior-most pair of developing lip papillae surrounding the mouth opening. Abbreviations: jw, presumptive jaws; pv, sclerotized plate (=preventral organ of the slime papilla segment); vo, ventral organ. Scale bars: 100 μm **(A)** and 50 μm **(B, C)**.

Although the slime papillae relocate anteriorly during embryonic development and take up a position on each side of the head, the anterior and posterior depressor muscles of each slime papilla retain their attachment to the corresponding ventral and preventral organs (Figure 9A–C). In contrast to the trunk segments, where the ventral and preventral organs are adjacent to each other, in the slime papilla segment they are separated by a pair of posterior lip papillae (arrowheads in Figure 9B, C). While the ventral organ of the slime papilla segment retains its position behind the developing mouth, the preventral organ is incorporated into the posterior wall of the mouth (Figure 9A, C). Interestingly, embryos of the neotropical Peripatidae, including *Principapillatus hitoyensis, Plicatoperipatus jamaicensis* and *Epiperipatus* sp. 2, show a sclerotized structure in the same position corresponding to the preventral organ of the slime papilla segment (Figure 10A–C).

## Discussion

### The issue of the “ventral organs”: Homonymy does not necessarily imply homology

The term ventral organs has been commonly applied to paired segmental thickenings in the ventral ectoderm of the onychophoran embryo [1,16,19,20,23,25]. These thickenings have been demonstrated to persist as midventral, segmental rudiments with an unknown function in adults [1,24]. However, our developmental data from *Euperipatoides rowelli* show that each pair of segmental thickenings gives rise to two paired structures, i.e., the ventral and preventral organs, both of which persist in adults. Although the paired nature of the ventral and preventral organs is mostly evident in embryos of late developmental stages, it is still recognisable in adults, as these structures are subdivided in two halves by a longitudinal, sclerotized internal ridge. Based on these findings, it seems clear that neither the ventral and preventral organs nor their anlagen correspond to the embryonic structures called “ventral organs” in chelicerates and myriapods [2,20,31,32]. While the ventral and preventral organs represent two paired structures per segment that persist in the epidermis of adult onychophorans, the homonymous “ventral organs” of chelicerates and myriapods arise as a single pair of vesicles per segment that are incorporated into each developing ganglion and disappear completely during development [2,17,19,20,31,32].

Moreover, in contrast to the “ventral organs” of chelicerates and myriapods, neither the ventral and preventral organs of onychophorans nor their embryonic anlagen arise as segmental ectodermal invaginations. The only invaginations of the ventral ectoderm in the onychophoran embryo are the anlagen of the hypocerebral organs in the antennal segment, which entirely lose their connection to the epidermis during development and become associated with the ventral surface of the brain [17,23-25]. The adult hypocerebral organs might be neurosecretory glands, homologous to the corpora allata of insects [21,22,34]. However, their serial homology to the ventral and preventral organs in onychophorans is uncertain [16] and there is no evidence for their homology to the embryonic “ventral organs” of chelicerates and myriapods, as they show an entirely different structure and fate [1]. Thus, neither the hypocerebral organs nor the ventral and preventral organs of onychophorans are likely to be homologous to the “ventral organs” of chelicerates and myriapods.

### Expression of *Delta* and *Notch* correlates with the embryonic origin of the ventral and preventral organs

Our gene expression data revealed two paired domains of *Delta* and *Notch* homologs in each embryonic segment of *Euperipatoides rowelli*, corresponding to regions that give rise to the ventral and preventral organs in this species. Exactly like the ventral and preventral organs, the posterior paired domain (corresponding to the anlagen of the ventral organ) is larger than the anterior domain (corresponding to the anlagen of the preventral organ). A similar pattern of expression was revealed in two previous studies (Figure 4B in [35]; Figure 4C in [36]) of the closely related species *Euperipatoides kanangrensis*. However, at the time when these studies [35,36] were carried out, the striking spatio-temporal correlation of this expression pattern with the paired anlagen of the ventral and preventral organs was unknown, and so the authors assumed an exclusive function of *Delta* and *Notch* as proneural genes in these regions of the developing embryo. In our view at least two arguments speak against this assumption. First, most neurons have already been segregated from the neuroectoderm at stages when *Delta* and *Notch* are expressed in two paired domains [1,17,35,36]. Second, the characteristic pattern of their domains, with a smaller anterior and a larger posterior pair, does not correspond to any known neural structure in onychophorans [10,11,29,37-39]. We therefore suggest that the double-paired *Delta* and *Notch* domains specify regions of the ectoderm that give rise to the ventral and preventral organs rather than to the neurogenic tissue in the onychophoran embryo.

It is well known that apart from functioning as “proneural genes” [40,41], *Delta* and *Notch* (Notch/Delta signalling) are involved in many other developmental processes, including cell death, cell division, and endocycle (DNA replication without an intervening mitosis) [42-46]. We have shown here that cell death and numerous cell divisions occur in the anlagen of the ventral and preventral organs in *Euperipatoides rowelli*. In addition, there is evidence that most of the cells in their anlagen might enter endocycle to increase biosynthetic activity [18]. Therefore, it might well be that in this case the genes *Delta* and *Notch* govern these processes in the anlagen of the preventral and ventral organs rather than being involved in neurogenesis, as suggested previously [35,36].

### The ventral and preventral organs serve as attachment sites for the ventral limb depressor muscles

Although the function of the ventral and preventral organs in adult onychophorans has remained unknown, they were affiliated with the nervous system, as they were thought to be connected to the nerve cords via a pair of cell strands [16,24]. Our data indeed show that each median commissure (as well as each ring commissure and each leg nerve) is accompanied by “glial” cells that might give the impression of cellular strands linking the ventral/preventral organs to each nerve cord. However, our detailed examination of complete series of histological and Vibratome sections, in conjunction with light, fluorescent and confocal microscopy, revealed no connections between the median commissures (which comprise neural tissue) and the ventral and preventral organs (which belong to the epidermis). Instead, they are clearly separated from each other by a thick layer of extracellular matrix and several layers of musculature and this spatial separation has also been reported from the onychophoran embryo (Figure 11C in ref. [1]).

In addition, we have shown herein that bundles of tracheal tubes are commonly associated with the ventral and preventral organs and they usually take a course towards each nerve cord by following the median commissures. This suggests that previous authors [16,24] might have misinterpreted the cells associated with tracheal tubes, the only cellular structures that pass through the extracellular matrix in onychophorans, as tissue connections between the nerve cords and the ventral organs. Moreover, from the perspective of functional morphology, a cellular bridge between the nerve cords and the ventral and preventral organs is unlikely to exist in Onychophora for two reasons: First, most median commissures pass to the contralateral side in regions in which the ventral and preventral organs are lacking and, second, the ventral and preventral organs are segmental structures, whereas the median commissures are repeated in a non-segmental fashion along the body [1,10,11,29]. Due to this lack of a corresponding spatial relationship between the ventral and preventral organs and the median commissures, it is unlikely that the ventral and preventral organs are in any way related to the nervous system.

Our data instead suggest that these structures are associated with the onychophoran leg musculature. According to our findings, the onychophoran ventral and preventral organs consist of epidermal cells that are covered by a thick cuticle, which forms hollow, sclerotized internal ridges. These ridges resemble typical apodemes of onychophorans and arthropods [12,47]. Furthermore, phalloidin- rhodamine labelling revealed that the anterior leg depressor muscles (see ref. [9] for the nomenclature) attach to each preventral organ, whereas the posterior leg depressor muscles connect to each ventral organ, which also holds true for the depressor muscles of the slime papillae. The presence of two leg depressor muscles might explain why the ventral and preventral organs comprise two paired rather than a single paired structure in each leg-bearing segment. Based on these findings, we suggest that the ventral and preventral organs of onychophorans serve as attachment sites for the paired, extrinsic limb depressor muscles.

## Conclusions

According to our findings, the ventral and preventral organs are a common feature of onychophorans, as they occur in representatives of Peripatidae and Peripatopsidae. During development, they arise from segmental thickenings of the embryonic ectoderm and persist in adults as sclerotized structures that serve as attachment sites for segmental limb depressor muscles. Whether the ventral and preventral organs are a derived feature of Onychophora or whether they are remnants of sclerotized, vaulted body rings described from fossil lobopodians [48] – putative stem-lineage representatives of Onychophora, Tardigrada, Arthropoda and/or Panarthropoda [49,50] – is open for discussion.

## Methods

### Specimens

Specimens of five species of Peripatidae and three species of Peripatopsidae were obtained from leaf litter and rotted logs at different localities (Table 1). While the anatomy of the ventral and preventral organs and associated structures was studied in all eight species of onychophorans, embryonic development was analysed only in the peripatopsid *Euperipatoides rowelli*. For this purpose, the animals were kept in plastic jars with perforated lids at 17°C as described previously [51]. Adult females were anaesthetised with chloroform vapour for 10–20 s and the reproductive tracts were dissected and transferred to dishes containing physiological saline [52]. After dissecting the embryos from the uteri, the embryonic membranes were removed manually using two forceps. The embryos were then fixed overnight in 4% paraformaldehyde (=PFA) in phosphate-buffered saline (=PBS; 0.1 mol/L, pH 7.4) and staged according to Walker and Tait [53] with the following modification. We classified stage V embryos more restrictively using the following characteristic features: (1) cerebral grooves (=anlagen of the hypocerebral organs) appear as longitudinal slits in the middle of each antennal lobe, and (2) the early anlagen of the last (15^th^) pair of walking legs have already formed. After staging, the embryos were either washed in PBS and used immediately for histochemical and immunocytochemical experiments or dehydrated in a graded methanol series and stored at -20°C for subsequent gene expression studies.

**Table 1:** Species of onychophorans studied and corresponding locality data

**Peripatidae**	
*Epiperipatus* sp. 1	Reserva Particular do Patrimônio Natural Estação Ambiental de Peti, 43°22′02.16224'' W, 19°53′33.44741'' S, 760 m, municipality of São Gonçalo do Rio Abaixo, Minas Gerais, Brazil
*Epiperipatus* sp. 2	PCH Porto das Pedras, 52°32′33.64'' W, 19°28′44.31'' S, 360 m, Mato Grosso do Sul state, municipality of Chapadão do Sul, Brazil
*Epiperipatus biolleyi* (Bouvier, 1902)	Los Juncos, 10°01′27.62'' N, 83°56′30.26'' W, 1760 m, Cascajal de Coronado, Province of San José, Costa Rica
*Principapillatus hitoyensis* Oliveira *et al.*, 2012	Reserva Biológica Hitoy Cerere, 09°40′21.56'' N, 83°02′36.97'' W, 300 m, Province of Limón, region of Talamanca, Costa Rica
*Plicatoperipatus jamaicensis* (Grabham & Cockerell, 1892)	Ecclesdown, eastern slope of John Crow Mountains, eastern coast of the island, Jamaica (more precise data unavailable)
**Peripatopsidae**	
*Euperipatoides rowelli* Reid, 1996	Tallaganda State Forest, 35°26′ S, 149°33′ E, 954 m, New South Wales, Australia
*Metaperipatus blainvillei* (Gervais, 1837)	A forest near Lago Tinquilco, 39°09′ S, 71°42′ W, 815 m, IX Region de la Araucania, Chile
*Metaperipatus inae* Mayer, 2007	Forest near Contulmo, 38°01′ S, 73°11′ W, 390 m, VIII Region del Biobio, Chile

### Stereomicroscopy and scanning electron microscopy

Adult specimens of all eight species studied (Table 1) were preserved in 70% ethanol and their ventral body surface was analysed with a stereomicroscope (Leica WILD M10, Leica Microsystems, Wetzlar, Germany) equipped with a digital camera (PCO AG SensiCam, Kelheim, Germany). For scanning electron microscopy, specimens of *Epiperipatus biolleyi, Principapillatus hitoyensis, Plicatoperipatus jamaicensis, Metaperipatus blainvillei* and *Metaperipatus inae* were fixed in 4% formaldehyde in PBS at room temperature. After several washes in water, the specimens were cut into suitable pieces, dehydrated in an ethanol series, dried in a CPD 030 Critical Point Dryer (BAL-TEC AG, Balzers, Liechtenstein), coated with gold in a SCD 040 Sputter Coater (BALZERS UNION, Balzers, Liechtenstein), and examined in a Quanta 200 Scanning Electron Microscope (FEI, Hillsboro, Oregon, USA). Embryos were dissected from some specimens after fixation and prepared in the same way as the body pieces from adults. Freshly moulted skins obtained from living specimens of *Principapillatus hitoyensis, Metaperipatus blainvillei* and *Metaperipatus inae* were spread on water surface according to Holliday [54], dehydrated in an ethanol series and processed further for scanning electron microscopy as described for the entire specimens.

### Histology, Vibratome sectioning and light and confocal laser-scanning microscopy

For histological studies, specimens of *Metaperipatus blainvillei* were fixed in Bouin’s fluid as described previously [55,56]. The specimens were dehydrated in an ethanol series, incubated in methylbenzoate and butanol, and embedded in Paraplast Tissue Embedding Medium (Kendall, Mansfield, MA, USA). Complete series of 5–7 μm thin sections were made with steel blades on a microtome (Reichert-Jung, 2050-supercut, Reichert Inc., Buffalo, NY, USA) and the sections were stained using Heidenhain’s [57] Azan staining method. The sections were then mounted on glass slides in Malinol and analysed under a light microscope (Leica Leitz DMR, Leica Microsystems) equipped with a digital camera (PCO AG SensiCam).

For Vibratome sectioning, specimens of *Metaperipatus blainvillei*, *Euperipatoides rowelli, Principapillatus hitoyensis* and *Epiperipatus* sp. 2 were cut in pieces and fixed overnight in 4% PFA in PBS at room temperature. The samples were then washed in several changes of PBS and either processed immediately or kept for several weeks in PBS containing 0.05% sodium azide at 4°C. Two embedding media were used for different body parts: (1) 6% agarose at 60°C, which was cooled down to room temperature as described previously [11], and (2) a 4:1 mixture of albumin/gelatine (3.75 g of albumin [Sigma-Aldrich, St. Louis, MO, USA, Grade II] in 10 ml distilled water; 0.5 g gelatine [Sigma-Aldrich, Type A] in 2.5 ml distilled water); the albumin/gelatine blocks were then fixed overnight in 10% PFA at 4°C and washed for 30 min in PBS at room temperature. The agarose and albumin/gelatine blocks were trimmed and sectioned into series of 100–200 μm thin sections with steel blades on a Vibratome (Vibratome Company, Saint Louis, USA). For morphological analyses of tracheal tubes, the sections were mounted on glass slides in 70% glycerine in PBS, covered with coverslips and imaged under a light microscope (Leica Leitz DMR) equipped with a digital camera (PCO AG SensiCam). For studies of myo- and neuroanatomy, the sections were processed for histochemistry and immunohistochemistry as described below, mounted on glass slides in Vectashield Mounting Medium (Vector Laboratories, Burlingame, CA) and analysed with the confocal laser-scanning microscopes Leica TCS STED (Leica Microsystems) and Zeiss LSM 510 META (Carl Zeiss MicroImaging GmbH, Jena, Germany).

### DNA labelling and detection of fragmented DNA or cell death

Embryos of *Euperipatoides rowelli* stored in methanol were rehydrated in PBS and either labelled with one of the DNA-selective fluorescent dyes (Hoechst [=Bisbenzimide, H33258; Sigma-Aldrich; 1 μg/ml in PBS], SYBR^®^ Green [Invitrogen, Carlsbad, CA, USA; 1:10,000 in PBS], Propidium Iodide [Roth, Karlsruhe, Germany; 1:3,000 in PBS], or RedDot^TM^2 [Biotium, Hayward, CA, USA; 1:250 in PBS]) for 1 h or used for the detection of fragmented DNA or cell death in conjunction with DNA labelling, as described previously [1]. For this purpose, the embryos were incubated in 0.1 mol/L sodium citrate (pH 6.0) for 30 min at 70°C and rinsed in PBS containing 1% Triton1 X-100 (=PBS-TX; Sigma-Aldrich). Detection of apoptotic cells was carried out with the *in situ* Cell Death Detection Kit, TMR red (Roche, Mannheim, Germany). The embryos were placed in equilibration buffer for 10 min at room temperature and the buffer was replaced with label solution (450 μl) and enzyme solution (50 μl) according to the manufacturer’s protocol. After incubation on a nutator (3 h at 37°C), the embryos were counterstained with Bisbenzimide (Sigma-Aldrich) and mounted in Vectashield Mounting Medium (Vector Laboratories). For negative controls, the embryos were treated in the same way but without adding the enzyme. These embryos showed no nuclear labelling. For positive controls, the embryos were treated with DNase I recombinant, RNase-free (Roche), prior to detection of cell death. In these embryos, all nuclei were labelled. All embryos were analysed with the confocal laser-scanning microscopes Leica TCS STED (Leica Microsystems) and Zeiss LSM 510 META (Carl Zeiss MicroImaging GmbH) as described for the Vibratome sections.

### Histochemistry and immunocytochemistry

Histochemical and immunocytochemical experiments on embryos and Vibratome sections of adult specimens of *Euperipatoides rowelli* were carried out as described previously [1,17,18,58,59]. Different fluorescent dyes and antisera were used either separately or in combination. As a general marker of neural structures, we used a monoclonal anti-acetylated α-tubulin antibody (Sigma-Aldrich; diluted 1:500 in PBS). As a mitosis marker, we applied a polyclonal anti-phospho-histone H3 antibody (=α-PH3; Upstate, Temecula, CA, USA; diluted 1:500 in PBS). For f-actin labelling, Vibratome sections and embryos were incubated overnight at room temperature in a solution containing phalloidin-rhodamine (Invitrogen) as described previously [1]. Some Vibratome sections and embryos were counterstained with one of the DNA-selective fluorescent dyes described in the section “DNA labelling and detection of fragmented DNA or cell death”. In addition, to reveal “glial” cells, nerve cords were dissected from adult specimens of *Euperipatoides rowelli*, fixed overnight at room temperature in 4% PFA, rinsed several times in PBS and labelled with one of the DNA-selective dyes as described above. The Vibratome sections and embryos were mounted on glass slides or between two coverslips either in Vectashield Mounting Medium or in Vectashield Hard Set Mounting Medium (Vector Laboratories). The nerve cords were dehydrated in an ethanol series and mounted between two coverslips in methyl salicylate. All samples were analysed with the confocal laser-scanning microscopes Leica TCS STED (Leica Microsystems) and Zeiss LSM 510 META (Carl Zeiss MicroImaging GmbH).

### Gene expression studies

RNA was isolated from embryos of *Euperipatoides rowelli* using TRIzol^®^ Reagent (Invitrogen). Library preparation and assembly of the embryonic transcriptomes were performed as described by Hering et al. [60]. To identify the *Er-Delta* and *Er-Notch* homologs, local tBLASTn searches were performed with the corresponding sequences from a closely related species of Onychophora, *E. kanangrensis*[35,36]. The identified *Er-Delta* and *Er-Notch* sequences [GenBank accession numbers: KF322113 and KF322114, respectively] were used to design specific primers and to amplify the *Er-Delta* and *Er-Notch* fragments. The PCR products were cloned into the *Escherichia coli* strain KJ100 using pGEM^®^-T Vector System (Promega Corporation, Madison, WI, USA). Each vector was linearized and transcribed *in vitro* using DIG RNA Labelling Kit SP6/T7 (Roche, Mannheim, Germany).

For *in situ* hybridization, the embryos were rehydrated in PBST (PBS + 0.1% Tween-20; 5 min each). Pre-hybridization was carried out for 6 h at 60°C, after which the probe was added to the embryos and incubated overnight at 60°C. Excess probe was removed by several rinses in hybridization buffer, saline-sodium citrate buffer + 0.1% Tween-20 and PBST, after which the embryos were incubated for 3 h in a blocking solution (10% normal goat serum in PBST) at room temperature. The embryos were then incubated with the Anti-DIG-AP antibody (Roche; diluted 1:1000 in blocking solution) overnight at 4°C. After several washes in PBST, a NBT/BCIP staining solution (Roche) was added to the embryos and the staining reaction was kept in the dark. The embryos were checked every 10 to 15 min under a dissection microscope in dimmed light until staining was evident and the staining reaction was stopped by several washes with PBST. To denature the alkaline phosphatase, the embryos were post-fixed in 4% paraformaldehyde, transferred into 1.5 ml reaction tubes and stored at 4°C. After staining, embryos were either analysed under a light microscope (Leica Leitz DMR, Leica Microsystems) equipped with a digital camera (PCO AG SensiCam) or counterstained with the DNA-selective dye Bisbenzimide as described above (see the section “DNA labelling and detection of fragmented DNA or cell death”) and analysed with the confocal laser-scanning microscope Zeiss LSM 510 META (Carl Zeiss MicroImaging GmbH).

### Image processing

Confocal image stacks were processed with Leica AS AF v2.3.5 (Leica Microsystems) and Zeiss LSM IMAGE BROWSER v4.0.0.241 (Carl Zeiss MicroImaging GmbH). Optimal quality light, confocal and scanning electron micrographs were achieved by using Adobe (San Jose, CA, USA) Photoshop CS5.1. Final panels were designed with Adobe Illustrator CS5.1 and exported in the Tagged Image File Format.

## Competing interests

The authors declare that they have no competing interests.

## Authors’ contributions

GM and ISO conceived and designed the work and wrote the first draft of the manuscript. GM, ISO and IS performed the experiments. All authors analysed the data and participated in the discussion of the results and the preparation of the final manuscript. All authors read and approved the final manuscript.

## Supplementary Material

Additional file 1 **Ventral and preventral organs in the genital segment in representatives of Peripatopsidae.** Scanning electron micrographs. Anterior is up in both images. (A) Detail of the genital opening in a juvenile of *Metaperipatus blainvillei*. (B) Detail of the genital opening in an adult specimen of *Metaperipatus inae*. Abbreviations: gp, genital pad; lg, leg; pv, preventral organ; vo, ventral organ. Scale bars: 50 μm (A, B). Click here for file

Additional file 2 **Bundles of tracheal tubes associated with preventral and ventral organs in adult onychophorans.** Through-light micrographs of Vibratome sections. Arrows indicate the position of the ventral and preventral organs, which are hard to distinguish due to the thickness of sections. Dorsal is up in both images. (A) Cross section of a region containing the ventral and preventral organs in *Principapillatus hitoyensis* (Peripatidae). Note the two conspicuous bundles of tracheae (arrowheads) in the vicinity of the ventral and preventral organs. (B) Sagittal section immediately lateral to the ventral and preventral organs in *Epiperipatus* sp. 2 (Peripatidae from Brazil). Note that bundles of tracheae open to the exterior in areas situated next to the ventral and preventral organs. Abbreviations: dp, dermal papilla; lg, leg; nc, nerve cord. Scale bars: 100 μm (A), and 200 μm (B). Click here for file

Additional file 3 **Bundles of tracheal tubes associated with nerve cords and commissures in *Metaperipatus blainvillei *** **(Peripatopsidae).** Through-light micrographs of Vibratome cross-sections from adult specimens. Dorsal is up and median is left in all images. (A) Dorso-lateral body region showing bundles of tracheae that follow the course of ring commissures into the nerve cord. (B) Ventro-lateral body region showing bundles of tracheae that follow the course of median commissures into the nerve cord. Note that some tracheal bundles open to the exterior on the ventral body surface close to the ventral midline (arrowhead). Abbreviation: bt, tracheal bundle; dp, dermal papilla; lg, leg; nc, nerve cord. Scale bars: 100 μm (A, B). Click here for file

Additional file 4 **“Glial” cells accompanying the leg nerves and median and ring commissures in *Principapillatus hitoyensis *** **(Peripatidae).** Confocal laser-scanning micrograph of a dissected nerve cord from an adult specimen labelled with the DNA marker SYBR^®^ Green. Lateral is left, anterior is up. Arrowheads point to the nuclei of single “glial” cells. Abbreviations: ln, anterior and posterior leg nerves; mc, median commissure; nc, nerve cord; rc, ring commissure. Scale bar = 50 μm. Click here for file

Additional file 5 **Arrangement of median commissures relative to ventral and preventral organs in *Euperipatoides rowelli *** **(Peripatopsidae).** Confocal laser-scanning micrographs. Anterior is left in both images. (A) Sagittal section of the anterior end of a newborn juvenile, double-labelled with an anti-acetylated α-tubulin antibody (green) and the DNA marker RedDot^TM^2 (blue). Dorsal is up. Note that the median commissures are not associated with the preventral and ventral organs but pass above the musculature layers from/to each nerve cord. Arrowheads point to single nerve fibres innervating the dermal papillae. (B) Arrangement of the median commissures along the body in an adult specimen labelled with an anti-acetylated α-tubulin antibody. Horizontal section of the ventral body wall. Doted circles indicate the position of the ventral and preventral organs in each segment. Note that the median commissures show no spatial relationship and lie out of register to the ventral and preventral organs. Abbreviations: lg, position of legs; mc, median commissure; ms, musculature; nc, nerve cord neuropil; pv, preventral organ; vo, ventral organ. Scale bars: 50 μm (A), and 100 μm (B). Click here for file

Additional file 6 **Muscles associated with the ventral and preventral organs.** Selected optic sections from a series of confocal laser-scanning micrographs (phalloidin-rhodamin labelling for f-actin; glow scale) showing the attachments sites (asterisks) of the anterior and posterior leg depressor muscles to the ventral and preventral organs in a newborn juvenile of *Euperipatoides rowelli* (Peripatopsidae). Abbreviations: pv, preventral organ; vo, ventral organ. Scale bar: 50 μm (in C for A–C). Click here for file
