# Supplementary figures and images for: The role of ventral and preventral organs as attachment sites for segmental limb muscles in Onychophora
Source: Front Zool. 2013 Dec 5;10:73. doi: 10.1186/1742-9994-10-73 (PMC3866996; doi:10.1186/1742-9994-10-73)

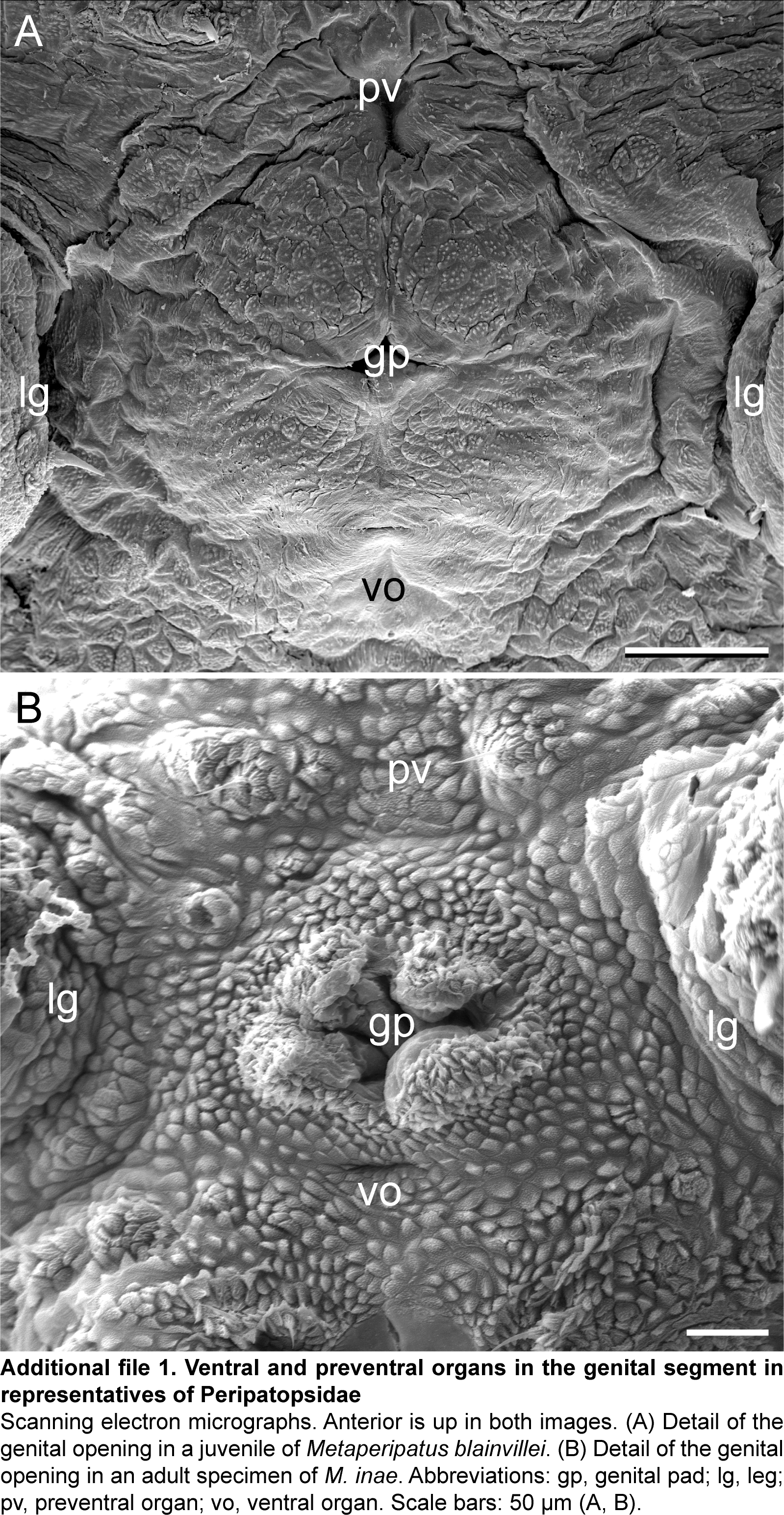

Supplement: Additional file 1 — Ventral and preventral organs in the genital segment in representatives of Peripatopsidae. Scanning electron micrographs. Anterior is up in both images. (A) Detail of the genital opening in a juvenile of Metaperipatus blainvillei. (B) Detail of the genital opening in an adult specimen of Metaperipatus inae. Abbreviations: gp, genital pad; lg, leg; pv, preventral organ; vo, ventral organ. Scale bars: 50 μm (A, B). [file 1742-9994-10-73-S1.tiff]

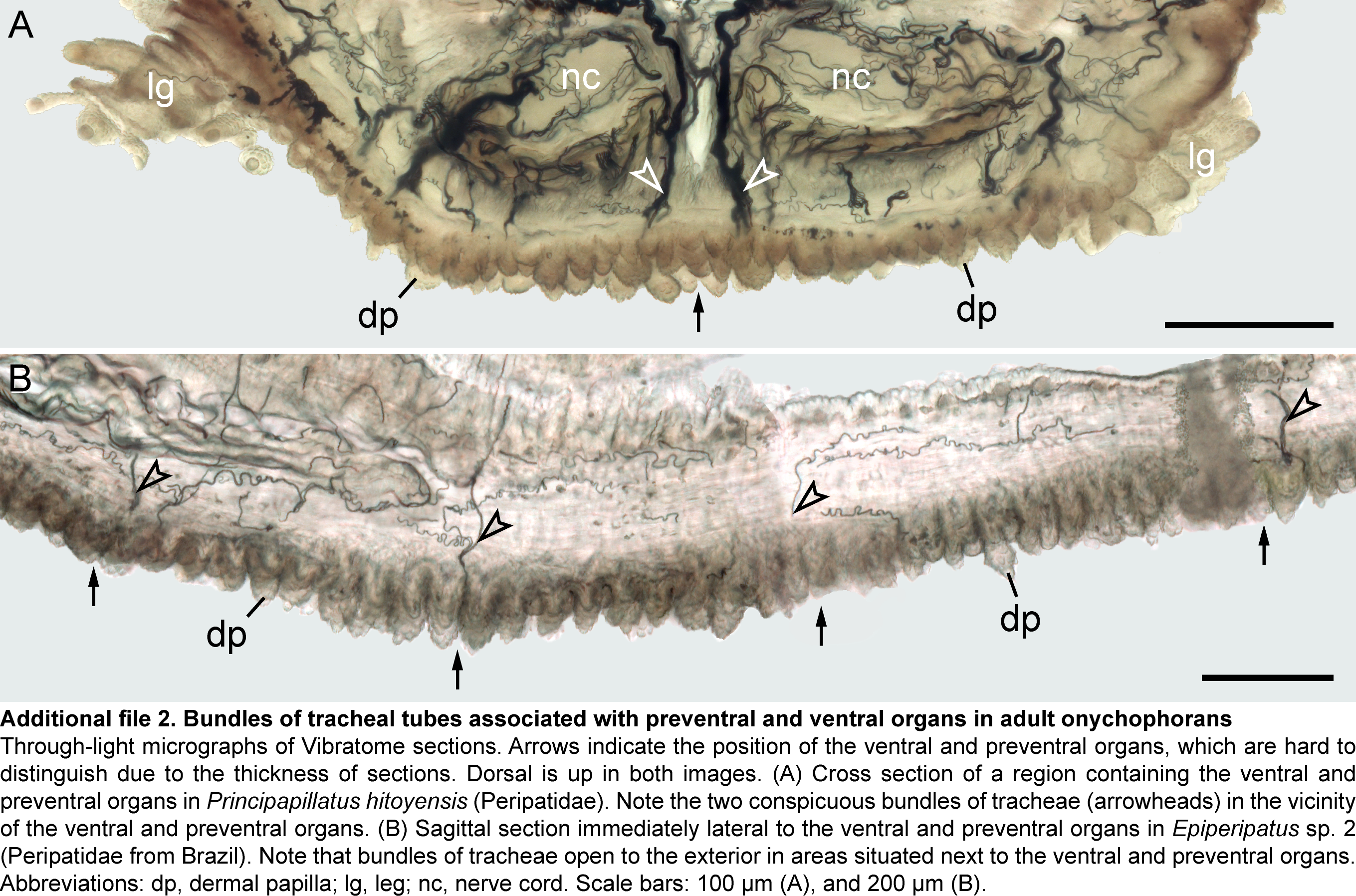

Supplement: Additional file 2 — Bundles of tracheal tubes associated with preventral and ventral organs in adult onychophorans. Through-light micrographs of Vibratome sections. Arrows indicate the position of the ventral and preventral organs, which are hard to distinguish due to the thickness of sections. Dorsal is up in both images. (A) Cross section of a region containing the ventral and preventral organs in Principapillatus hitoyensis (Peripatidae). Note the two conspicuous bundles of tracheae (arrowheads) in the vicinity of the ventral and preventral organs. (B) Sagittal section immediately lateral to the ventral and preventral organs in Epiperipatus sp. 2 (Peripatidae from Brazil). Note that bundles of tracheae open to the exterior in areas situated next to the ventral and preventral organs. Abbreviations: dp, dermal papilla; lg, leg; nc, nerve cord. Scale bars: 100 μm (A), and 200 μm (B). [file 1742-9994-10-73-S2.tiff]

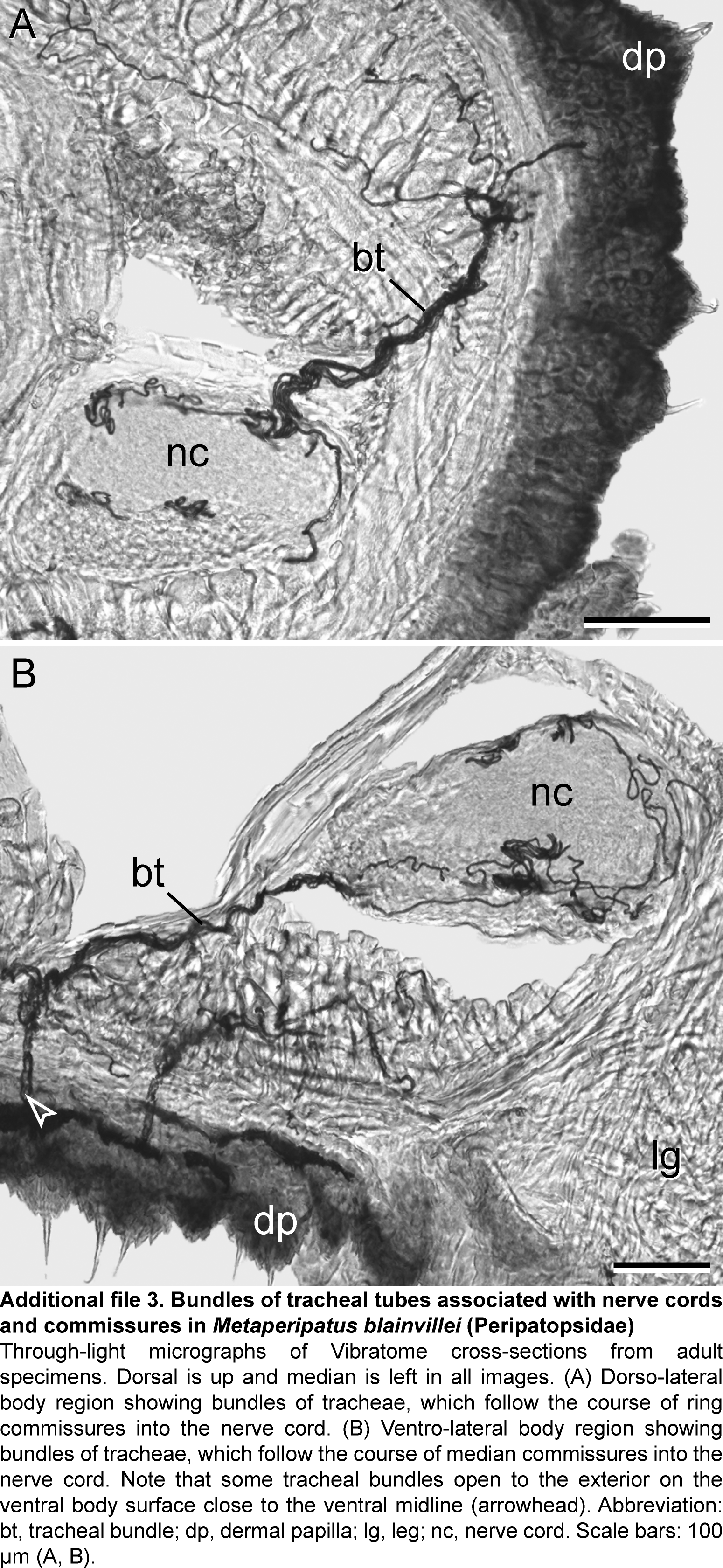

Supplement: Additional file 3 — Bundles of tracheal tubes associated with nerve cords and commissures in Metaperipatus blainvillei (Peripatopsidae). Through-light micrographs of Vibratome cross-sections from adult specimens. Dorsal is up and median is left in all images. (A) Dorso-lateral body region showing bundles of tracheae that follow the course of ring commissures into the nerve cord. (B) Ventro-lateral body region showing bundles of tracheae that follow the course of median commissures into the nerve cord. Note that some tracheal bundles open to the exterior on the ventral body surface close to the ventral midline (arrowhead). Abbreviation: bt, tracheal bundle; dp, dermal papilla; lg, leg; nc, nerve cord. Scale bars: 100 μm (A, B). [file 1742-9994-10-73-S3.tiff]

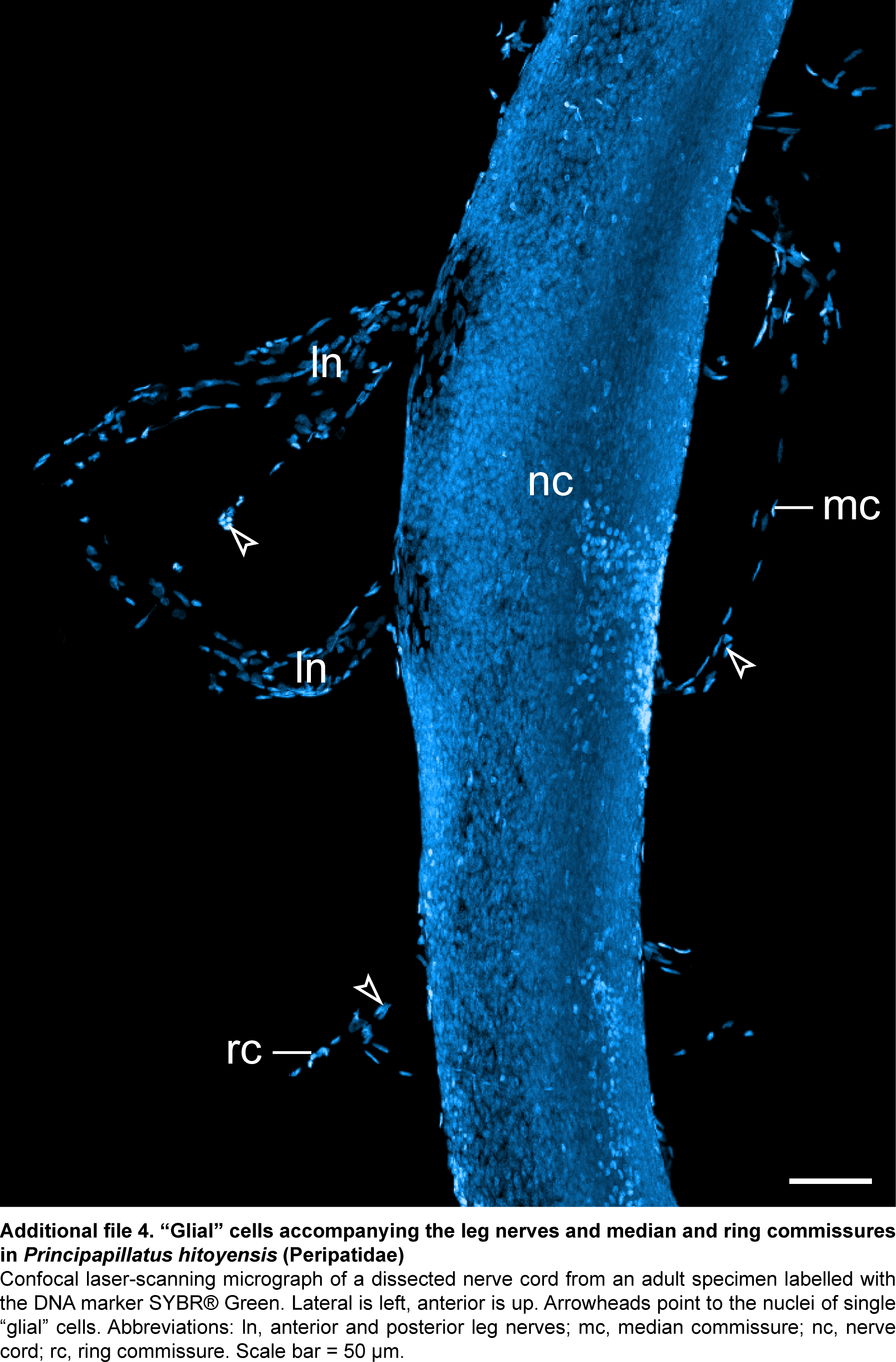

Supplement: Additional file 4 — “Glial” cells accompanying the leg nerves and median and ring commissures in Principapillatus hitoyensis (Peripatidae). Confocal laser-scanning micrograph of a dissected nerve cord from an adult specimen labelled with the DNA marker SYBR® Green. Lateral is left, anterior is up. Arrowheads point to the nuclei of single “glial” cells. Abbreviations: ln, anterior and posterior leg nerves; mc, median commissure; nc, nerve cord; rc, ring commissure. Scale bar = 50 μm. [file 1742-9994-10-73-S4.tiff]

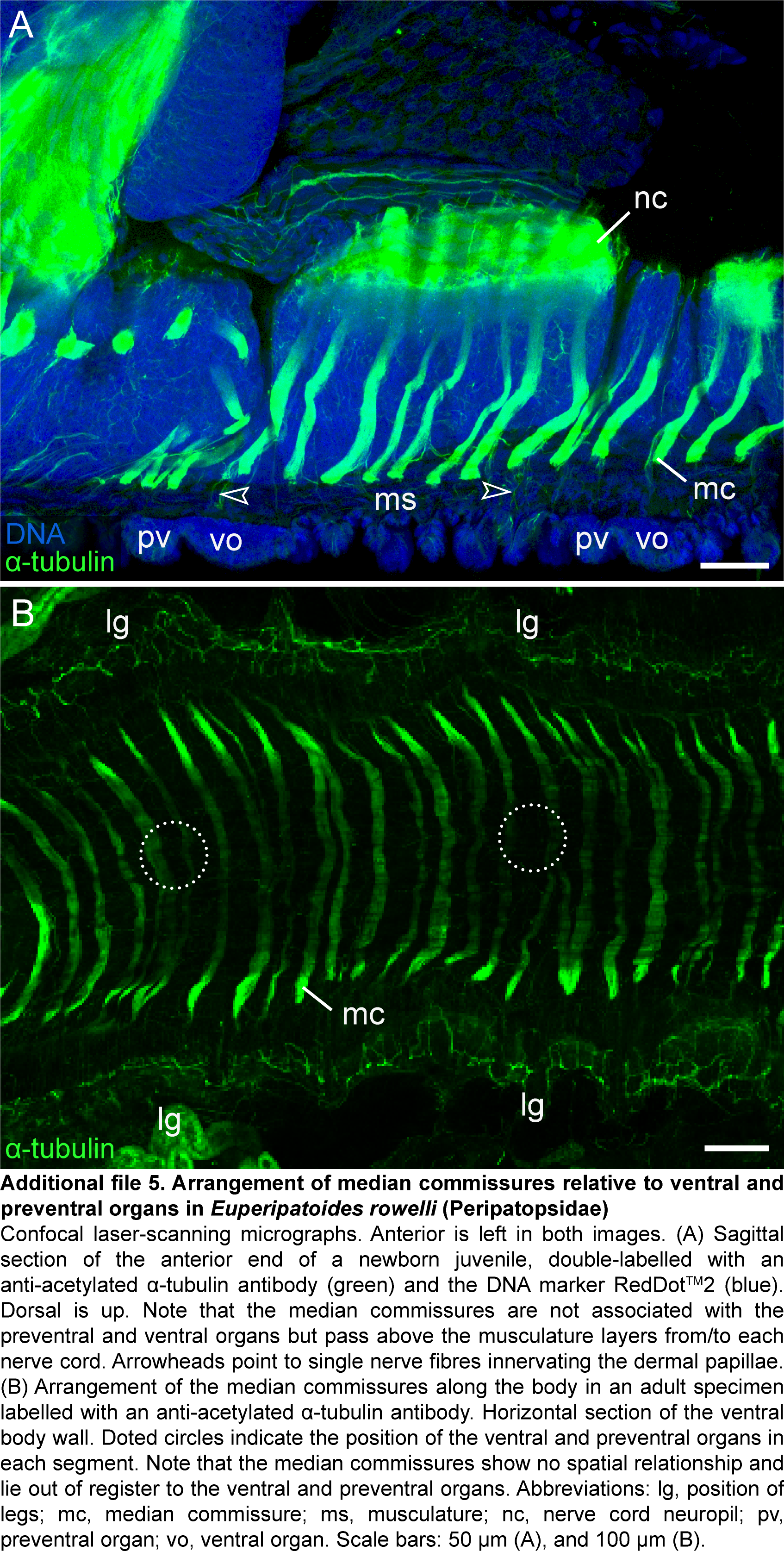

Supplement: Additional file 5 — Arrangement of median commissures relative to ventral and preventral organs in Euperipatoides rowelli (Peripatopsidae). Confocal laser-scanning micrographs. Anterior is left in both images. (A) Sagittal section of the anterior end of a newborn juvenile, double-labelled with an anti-acetylated α-tubulin antibody (green) and the DNA marker RedDotTM2 (blue). Dorsal is up. Note that the median commissures are not associated with the preventral and ventral organs but pass above the musculature layers from/to each nerve cord. Arrowheads point to single nerve fibres innervating the dermal papillae. (B) Arrangement of the median commissures along the body in an adult specimen labelled with an anti-acetylated α-tubulin antibody. Horizontal section of the ventral body wall. Doted circles indicate the position of the ventral and preventral organs in each segment. Note that the median commissures show no spatial relationship and lie out of register to the ventral and preventral organs. Abbreviations: lg, position of legs; mc, median commissure; ms, musculature; nc, nerve cord neuropil; pv, preventral organ; vo, ventral organ. Scale bars: 50 μm (A), and 100 μm (B). [file 1742-9994-10-73-S5.tiff]

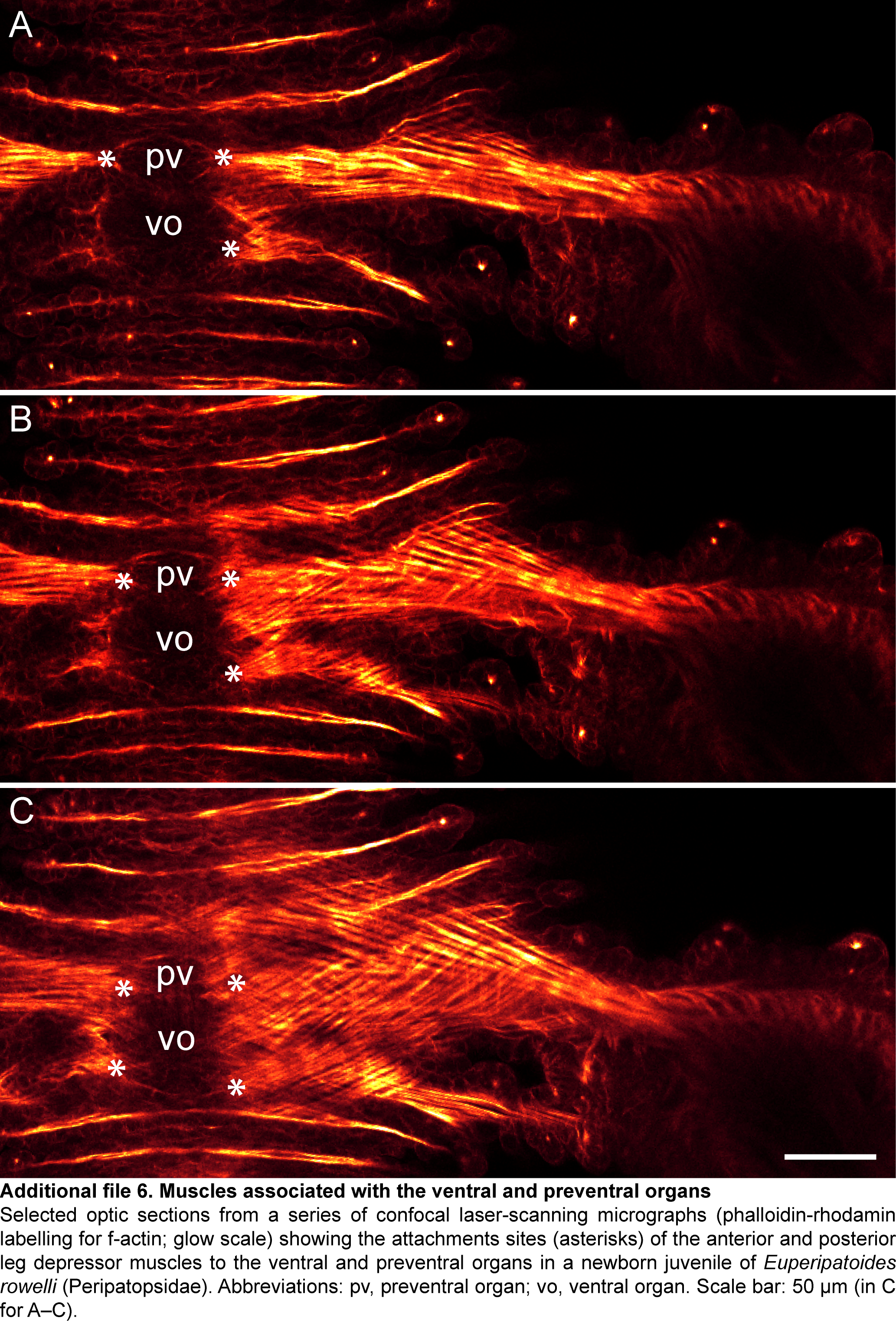

Supplement: Additional file 6 — Muscles associated with the ventral and preventral organs. Selected optic sections from a series of confocal laser-scanning micrographs (phalloidin-rhodamin labelling for f-actin; glow scale) showing the attachments sites (asterisks) of the anterior and posterior leg depressor muscles to the ventral and preventral organs in a newborn juvenile of Euperipatoides rowelli (Peripatopsidae). Abbreviations: pv, preventral organ; vo, ventral organ. Scale bar: 50 μm (in C for A–C). [file 1742-9994-10-73-S6.tiff]
